# New Sensors and Techniques for the Structural Health Monitoring of Propulsion Systems

**DOI:** 10.1155/2013/596506

**Published:** 2013-07-11

**Authors:** Mark Woike, Ali Abdul-Aziz, Nikunj Oza, Bryan Matthews

**Affiliations:** ^1^National Aeronautics and Space Administration, Glenn Research Center, Cleveland, OH 44135, USA; ^2^National Aeronautics and Space Administration, Ames Research Center, Moffett Field, CA 94035, USA

## Abstract

The ability to monitor the structural health of the rotating components, especially in the hot sections of turbine engines, is of major interest to aero community in improving engine safety and reliability. The use of instrumentation for these applications remains very challenging. It requires sensors and techniques that are highly accurate, are able to operate in a high temperature environment, and can detect minute changes and hidden flaws before catastrophic events occur. The National Aeronautics and Space Administration (NASA), through the Aviation Safety Program (AVSP), has taken a lead role in the development of new sensor technologies and techniques for the in situ structural health monitoring of gas turbine engines. This paper presents a summary of key results and findings obtained from three different structural health monitoring approaches that have been investigated. This includes evaluating the performance of a novel microwave blade tip clearance sensor; a vibration based crack detection technique using an externally mounted capacitive blade tip clearance sensor; and lastly the results of using data driven anomaly detection algorithms for detecting cracks in a rotating disk.

## 1. Introduction

The development of in situ measurement technologies and fault-detection techniques for the structural health monitoring of gas turbine engines is of major interest to NASA's Aviation Safety Program and the aeronautical community. The rotating components of modern gas turbine engines operate in severe environmental conditions and are exposed to high thermal and mechanical loads. The cumulative effects of these loads often lead to high stresses, structural deformities, cracks, and eventual component failure. Current structural health monitoring practices involve periodic inspections and schedule-based maintenance of engine components to ensure their integrity over the lifetime of the engine. However, these methods have their limitations, and failures are experienced leading to unscheduled maintenance and unplanned engine shutdowns. To prevent these failures and enhance aviation safety, the NASA Glenn Research Center has investigated new sensor technologies and techniques for the in situ structural health monitoring and detection of defects in gas turbine engines.

Much of the research effort to date has dealt with the development of low technology readiness level (TRL) structural health concepts with the intent of validating these concepts and transitioning them to higher TRLs for usage on actual aero engine hardware. In this study, microwave blade tip clearance and blade tip timing sensor technology is being investigated as a means of making high temperature noncontact structural health measurements in the hot sections of gas turbine engines. It is specifically being targeted for use in the high pressure turbine (HPT) and high pressure compressor (HPC) sections to directly monitor the structural health of the rotating components. The capability to make in situ health measurements is a need that has been identified by the aero engine community as blade damage in the HPT and HPC sections account for 12 percent of the inflight engine shutdown events and 32 percent of the damage events that caused engine removal for unscheduled maintenance [1].

Currently there are no off-the-shelf blade tip clearance sensors that are used in commercial turbine engines for in-situ structural health monitoring, as there are challenges with the sensors being able to operate in and survive the harsh high temperature environment. Microwave sensor technology is appealing in that it is accurate, it has the ability to operate at extremely high temperatures, and is unaffected by contaminants that are present in turbine engines. In addition to its use for structural health monitoring, this type of sensor also has parallel usage in improving engine performance through its use in active turbine tip clearance control schemes. As a means of better understanding the issues associated with the microwave sensors, a series of evaluation experiments were conducted to evaluate their performance on aero turbine engine like hardware.

Along with the development and evaluation of new types of blade tip clearance sensors, efforts have been placed into developing techniques that utilize these sensors for the structural health monitoring of engine components. The vibration based crack detection experiment that will be discussed involved introducing a notch to simulate a crack on a subscale turbine engine disk and monitoring its vibration response as the disk was rotated at speeds up to 12 000 rpm. The vibration response was characterized by using externally mounted capacitive blade tip clearance sensors to measure the combined disk-rotor system's whirl amplitude and phase during operation. Testing was performed on a clean undamaged baseline disk and a disk with a 50.8 mm long notch machined into the disk to simulate a crack. The responses were compared and evaluated against the theoretical models to investigate the applicability and success of detecting the notch.

And finally, in parallel with the vibration based crack detection investigation an experiment using data driven anomaly detection algorithms was undertaken as a means of evaluating whether these data mining techniques could be used to detect a fault or an anomaly in a rotating engine disk. In this experiment blade tip clearance data sets were acquired on an undamaged subscale engine rotor disk as it was operated at speeds up to 10 000 rpm. This baseline data was used to train three different data mining algorithms. Data was then acquired from a damaged disk with a known crack and the data mining algorithms were used to detect the presence of the crack.

This paper presents a summary of key results and findings obtained from three different structural health monitoring approaches that have been investigated. This includes evaluating the performance of a novel microwave blade tip clearance sensor; a crack detection technique using externally mounted blade tip clearance sensors; and lastly discussing the results of a data driven anomaly detection technique for sensing cracks in a rotating rotor disk.

## 2. Microwave Blade Tip Clearance/Blade Tip Timing Sensors

The ability to monitor the structural health of the rotating components, especially in the hot sections of turbine engines, is of major interest to aero community in improving engine safety and reliability [2]. In addition, the active control and minimization of the gap between the rotating turbine blades and the stationary case of gas turbine engines is being sought as a means of increasing engine efficiency, reducing fuel consumption, reducing emissions, and increasing engine service life [3]. The use of instrumentation for these applications in gas turbine engines requires sensors that are highly accurate and can operate in a high temperature environment. To address this need, microwave sensor technology is being investigated as a means of making high temperature non-contact blade tip clearance and tip timing measurements for use in structural health monitoring and active clearance control applications in turbine engines. This technology is appealing due to its high accuracy and its potential to operate at extremely high temperatures that are present in turbine engines. It is intended to use blade tip clearance to monitor blade growth and wear and blade tip timing to monitor blade vibration and deflection.

### 2.1. Sensor Background and Theory

NASA has worked with Radatec (now Meggitt Inc.) through the Small Business Innovation Research (SBIR) program for the development of microwave sensor technology for high temperature noncontact blade tip clearance and blade tip timing measurements. The initial development of the technology was accomplished through a phase II SBIR contract awarded in 2002. Further development of the technology was accomplished in 2004 through 2005 as part of NASA's Ultra Efficient Engine Technology (UEET) Program. A prototype first generation 5.8 GHZ system was delivered as part of a phase III SBIR commercialization contract in 2007 and a second generation 24 GHZ system along with upgraded electronics was delivered as part of subsequent follow-on contracts in 2009 and 2010, respectively.

**Figure 1:**
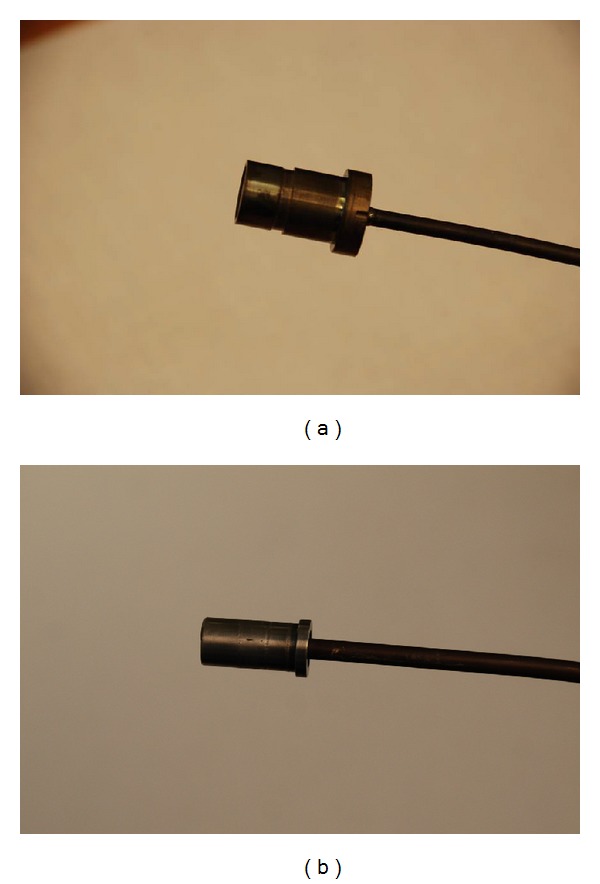
Microwave blade tip clearance probe (Meggitt). (a) 5.8 GHz probe. (b) 24 GHz probe.

The microwave blade tip clearance sensor operates essentially as a field disturbance device. The tip clearance probe contains both a transmitting and receiving antenna. The sensor emits a continuous microwave signal and measures the signal that is reflected off a rotating blade. The sensor measures the changes in the microwave field due to the blade passing through the field. The motion of the blade phase modulates the reflected signal and this reflected signal is compared to an internal reference. Changes in amplitude and phase directly correspond to the distance to the blade. The time interval of when the blade passes through the field is measured to provide blade tip timing. More detailed information on the sensor's theory of operation can be found in [4–6]. The microwave blade tip clearance probes are made of high temperature material and are designed to operate in temperatures up to 900°C. The first generation probes (Figure 1(a)) operate at 5.8 GHz and can measure clearance distances up to one-half the radiating wavelength which is 25 mm. The second generation probes (Figure 1(b)) operate at 24 GHz and can measure clearance distances up to 6 mm. The 5.8 GHz sensor is targeted for use on large rotating machinery such as land based power turbines or in the fan sections of aero gas turbine engines. The 24 GHz sensor is being targeted for use in smaller rotating machinery applications such as the turbine and compressor sections of aero engines. This technology has an ultimate goal of obtaining clearance measurement accuracies approaching +/−25 *μ*m. A frequency response of up to 5 MHz is typical, with up to 25 MHz being possible with this technology which lends itself for use in structural health monitoring applications for the measurement of blade deflection and vibration.

### 2.2. Experimental Results

NASA's primary goal is to demonstrate this microwave blade tip clearance sensor technology on an actual gas turbine engine in a relevant high temperature environment. However, the use of microwave sensors for this application is an emerging concept. Techniques on their use and calibration needed to be understood and developed. In addition, the microwave sensor's accuracy and ability to make blade tip clearance and deflection measurements had to be assessed prior to use on actual engine hardware. As a means of better understanding the issues associated with the microwave sensors, a series of experiments were conducted to evaluate the sensor's performance on aero engine type applications. A summary of these experiments and their results are as follows.

**Figure 2:**
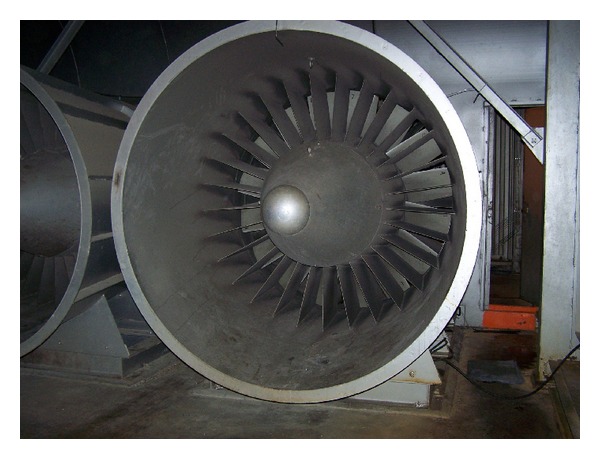
Axial vane fan located at the 10 × 10 SWT.

**Figure 3:**
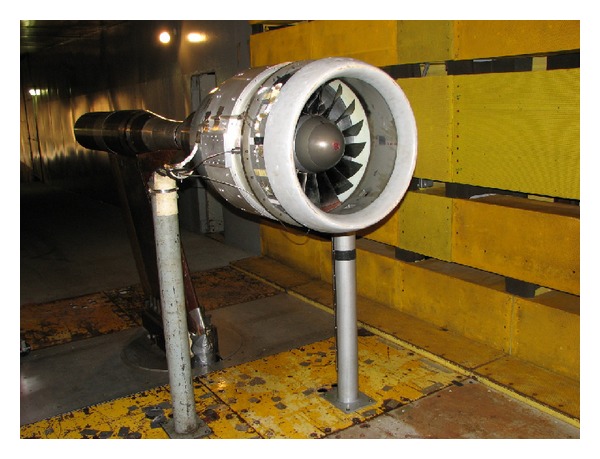
NASA Turbofan.

**Figure 4:**
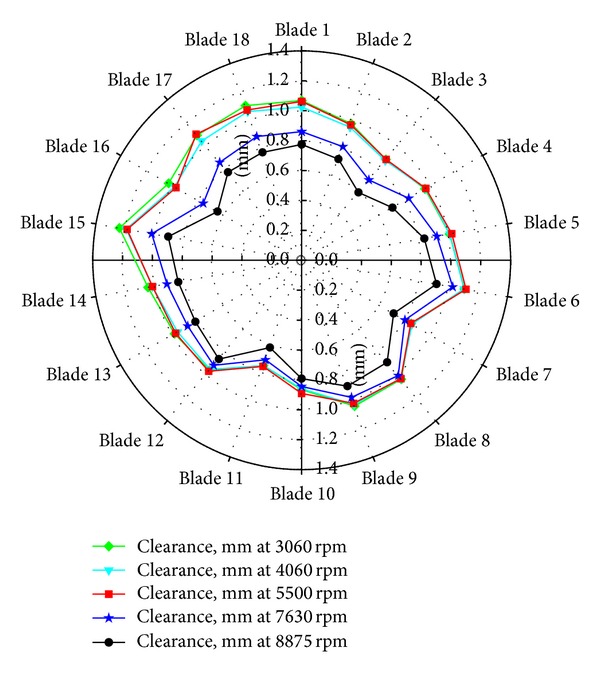
Blade tip clearance polar plot for probe #1, 90° location—NASA Turbofan experiment.

The first generation 5.8 GHz microwave sensors were used to a make blade tip clearance measurements on a large axial vane fan and a subscale NASA Turbofan. The purpose of the test on the large Axial Vane Fan (Figure 2) was to develop the infrastructure required for the calibration of the sensors and the techniques for their use in the field to make clearance measurements on large rotating machinery. The motivation behind their use on the NASA Turbofan (Figure 3) was to evaluate the first generation sensor's ability to acquire blade tip clearance data on an aero engine size test article and blades. Blade tip clearance data sets were acquired for several test runs of the NASA Turbofan. Data was acquired at a variable sampling rate that was synchronized to the fan's speed. Each measurement consisted of two revolutions of data with 10 000 samples taken per revolution.  Figure 4 shows the individual blade clearances measured for several fan speeds. It is clearly noted from the polar plot that the tip clearances decreased as the fan speed is increased.

This result is expected and is due to the growth and expansion of the turbofan's composite blades as the fan operates at higher speeds. An average decrease of 0.22 mm was observed as the rig speed was increased to 8 875 rpm. The change in clearance detected in this experiment was within the range predicted for these blades. In addition, the change in tip clearances measured by the microwave sensors was nearly identical to previously recorded values obtained with capacitive clearance sensors. In previous test entries, changes in tip clearances of up to 0.22 mm were also noted when the turbofan was operated over the same speed range. These two initial experiments served as test beds for the development of techniques and infrastructure required for the calibration of the sensors and successfully demonstrated the microwave clearance sensor's ability to make measurements on aero engine size hardware.

**Figure 5:**
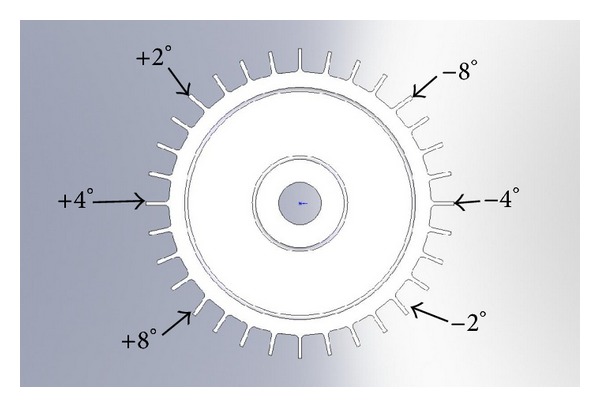
Simulated turbine disk with prebent blades for blade tip deflection testing.

The second generation 24 GHz sensors were used to make measurements on a 32 blade subscale turbine engine like disk and on an actual compressor stage from a small aero engine. These experiments were conducted in a laboratory environment using a calibration spin rig, a High Precision Spin Rig, and a High Temperature Spin Rig for the purposes of evaluating the second generation sensor's capability of making both clearance and timing measurements on small aero engine hardware. The 32 blade subscale engine disk (Figure 5) was manufactured to have 6 equally spaced blades that were bent at predefined angles or deflection distances. The purpose of this experiment was to evaluate the sensors ability to generate blade tip clearance and blade tip timing measurements simultaneously. During this experiment it was successfully demonstrated that the sensors were able to reliably make absolute measurements down to a clearance level of 100 *μ*m. This was an improvement over the previously demonstrated minimum measurement of 500 *µ*m made with the first generation sensors. This ability to measure very low clearance ranges is critical for use in closed loop clearance control applications. In addition, the sensor was able to simultaneously detect the minimum deflection of 0.70 mm (2 degrees) that was machined onto the disk. It is planned to further explore this capability by fabricating a disk that has smaller deflections in order to see the minimum deflection that can be reliably measured. 

The investigation using a compressor stage from a small aero engine was conducted for the purposes of calibrating the sensors for future use on a small compressor and evaluating their ability to measure blade tip clearances on actual engine hardware over the very low clearance ranges typically associated with aero engines. For this experiment clearances were successfully measured over a range from 0.10 to 1.50 mm with a maximum observed error of +/− 0.021 mm. These results were encouraging in that the sensor was able to make measurements on actual aero engine hardware over a relatively low clearance range within the desired accuracy of +/− 0.025 mm. However, it is acknowledged that these results were achieved in the ideal set-up of a laboratory and that the results may differ in an actual engine due to noise and other environmental effects. 

In summary, the evaluation testing that was accomplished on the microwave blade tip clearance technology has shown that sensors are a viable option for propulsion health monitoring and clearance control applications. Techniques for the calibration, integration, and use of the sensors to make measurements on aero engine hardware were successfully developed and both the first and second generation sensors have been successfully demonstrated on rotating machinery and aero engine hardware. A demonstration test of these sensors along with other advanced technologies on an engine ground test is being planned to occur in the 2013-2014 timeframe.

## 3. Vibration Based Crack Detection Technique

This crack-detection methodology involved introducing a notch on a simulated turbine engine disk and monitoring its vibration response as the disk was rotated at speeds up to 12 000 rpm. The vibration response was characterized by monitoring the disk-rotor system's whirl amplitude and phase during operation. The whirl amplitude and phase were derived from the blade tip clearance profile that was measured using externally mounted blade tip clearance sensors. This type of sensor was chosen as it represents the type of sensor that is most likely to be installed on a commercial engine due to its minimal installation impact, low overhead, and parallel benefits that it can bring to improve engine performance through its use in active turbine tip clearance control. The testing was performed on a clean undamaged baseline disk and on a damaged disk with a 50.8 mm long notch machined into the disk to simulate a crack. The experiment was conducted at the NASA Glenn Research Center's High Precision Spin Rig, a rig which is ideally suited for the development and validation of low technology readiness level (TRL) concepts before implementation on more expensive and complicated rotating machinery. A description of the High Precision Spin Rig, the baseline theory behind this technique, the experimental setup, and the results are discussed. 

### 3.1. High Precision Spin Rig Description

**Figure 6:**
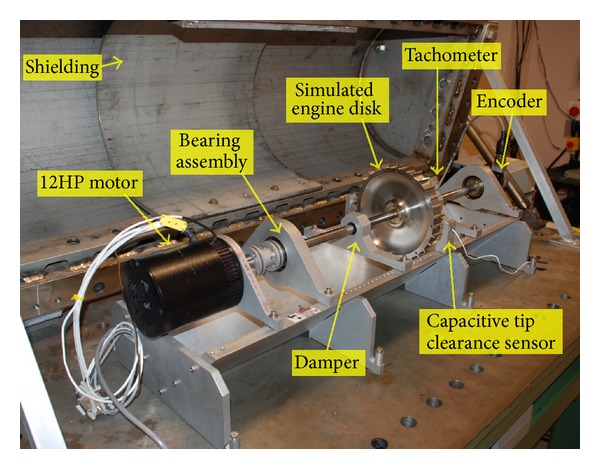
High Precision Spin Rig.

Figure 6 shows the Rotordynamics Laboratory's High Precision Spin Rig, which can accommodate simulated engine rotor disks of up to 235 mm in diameter. It has a stainless steel shaft with a length of 781 mm and diameter of 20 mm. The shaft is supported by precision contact ball bearings on each end and has adjustable dampers that were positioned along the length of the shaft for these experiments. An encoder mounted on the end of the shaft was used by the control system to provide closed-loop control of rig speed. A secondary optical tachometer was used to record the speed into the data system and to synchronize the data to the rig's rotation. A 12-hp custom-built, brushless direct-current (dc) motor was used to rotate the spin rig and the subscale engine disks at speeds up to 12 000 rpm.

**Figure 7:**
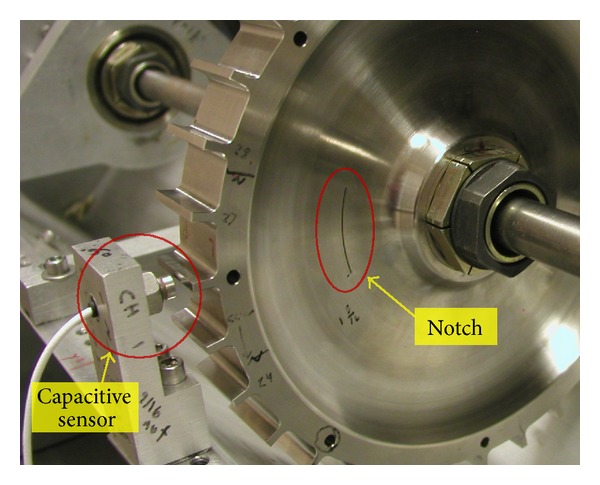
Capacitive blade tip clearance sensor and subscale turbine engine disk with notch.

The rig was set up to acquire two channels of radial blade tip clearance data from the simulated engine disk using capacitive displacement sensors (Figure 7). These sensors were developed as part of a NASA Small Business Innovation Research (SBIR) contract and were different from traditional capacitive sensors in that their operation was based on a dc offset technique instead of the typical modulation technique. A National Instruments System was used to acquire data from the capacitive displacement probes at a fixed sampling rate of 1 MHz. This system and its application software were delivered as part of the SBIR contract for the capacitive blade tip clearance sensors. The system used custom data acquisition and processing applications that were tailored for acquiring and processing data from the blade tip clearance sensors.

### 3.2. Experimental Theory and Setup

The theory behind the crack-detection methodology that was investigated was based on previous theoretical and experimental work performed by Abdul-Aziz et al. [7, 8], Gyekenyesi et al. [9, 10], and Haase and Drumm [11]. The goal of this experiment was to determine if the crack-detection methodology investigated in these earlier studies could be validated by using the spin rig to conduct tests on simulated engine rotor disks with a notch introduced to replicate a crack.

The detection methodology is based on monitoring the vibration response of rotating disks to determine if a crack has developed. The theory implies that a defect, such as a crack, creates minute deformations in the disk as it is rotated. The deformation, in turn, creates a speed-dependent shift in the disk's center of mass. It was theorized that this shift could be detected by analyzing the vibration whirl amplitude and phase as measured by the blade tip clearance profile. The system's behavior was modeled after a 2-degree-of-freedom Jeffcott rotor. The model predicts that the vibration amplitude peaks when a clean, undamaged disk goes through the first critical speed but heads to a lower steady-state value as the speed is increased above the critical speed. Correspondingly, the phase shifts 180° when going through the first critical speed and then stabilizes to a steady-state value as the speed is increased past critical. At this point, the rotor is rotating about the combined system's center of mass and has stabilized. However, as the speed of a cracked disk is increased, centrifugal forces open the crack. This, in turn, deforms the disk and shifts its center of mass. At speeds above critical, the crack-induced shift in the disk's center of mass starts to grow and dominate the overall system's vibration response. The modeling predicts that, instead of heading toward a steady-state value, the vibration amplitude will change as a second-order function of rotational speed as it is increased beyond the first critical speed. This can be detected by analyzing the vibration response of the disk-rotor system, particularly the amplitude and phase of the first harmonic, as the system is operated over a range of speeds.

The testing approach used in this experiment was to spin a subscale engine disk over a speed range from 0 to 12 000 rpm and simultaneously record its vibration response. The vibration response was acquired as previously described using capacitive displacement probes to measure the blade tip clearance profile. Two subscale simulated engine turbine disks were tested. The first disk was undamaged and was used to acquire the baseline vibration response data. The disk had an outside diameter of 235 mm, a bore thickness of 25.4 mm, and an outside rim thickness of 31.75 mm. The thinnest portion of the disk's web was 2.54 mm. Thirty-two teeth to simulate blades were evenly spaced around the circumference of the disk. Each simulated blade had a cross section of 31.75 mm by 3.30 mm and a height of 8.38 mm. The disk was made of a nickel base alloy, Haynes X-750 (Haynes International, Inc.) and had a weight of 4.88 kg.

The damaged disk is shown in Figure 7. It was identical to the baseline disk with the exception that a 50.8 mm long notch had been introduced in its mid-span region to imitate a crack in the disk. The mid-span region was selected since prior finite-element analysis had shown that this area experienced high stress levels during operation. The data acquired from this disk were compared with the data from the undamaged baseline disk to determine if the simulated crack could be detected by analyzing the vibration response. Supportive analytical calculations were made using finite-element analysis to complement the experimental work and determine the expected radial growth of the disk. The finite-element analysis predicted a radial growth on the order of ~0.075 mm at the highest operating speed, 12 000 rpm. This was within the detection limit of the blade-tip-clearance instrumentation. However, it should be noted that the major effect that was to be monitored was how the notch changed the combined center of mass of the disk-rotor system and its vibration amplitude and phase, not the radial blade tip growth of the disk.

### 3.3. Experimental Results

The experimentation consisted of operating the baseline and notched disks at several speed profiles and recording their vibration responses using the capacitive blade tip clearance sensors. The first harmonic component of the tip-clearance profile for each revolution was then analyzed to determine if the amplitude and phase exhibited the expected characteristics associated with a crack in the disk. Approximately 15 test runs were conducted for each disk. A typical test run or cycle consisted of ramping up the disk's speed from 0 rpm to a predefined maximum speed, remaining on condition for 0 to 60 s, then ramping back down to 0 rpm. Blade tip clearance data were acquired over the entire cycle. Data sets were acquired at ramp rates of 60 and 100 rpm/s for peak operating speeds of 5 000, 10 000, and 12 000 rpm. In addition, complex mission profiles were conducted where the speed was ramped to various power levels in an attempt to simulate the rigorous loading conditions that an engine would experience during a typical mission.

**Figure 8:**
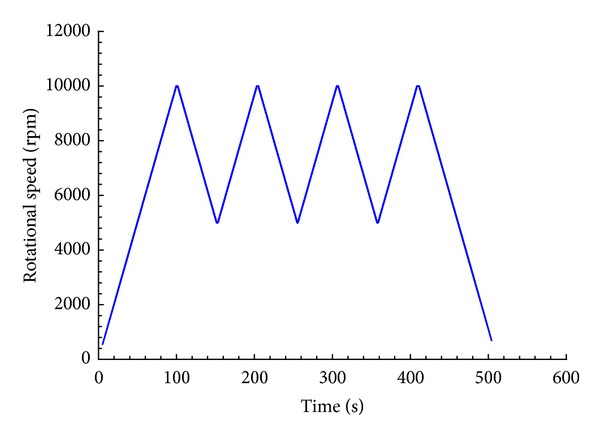
Simulated mission profile.

The run profile presented in this paper is shown in Figure 8. It is typical of the profiles that were used in the investigation. For this test case, the speed of the disk was increased from 0 to 10 000 rpm at a ramp rate of 100 rpm/s, cycled between 10 000 and 5 000 rpm for three cycles, then ramped down from 10 000 to 0 rpm. This was done to simulate a mission profile from start-up to full power and to observe the effects of cyclical loading on the disks. This ramping up and down also maximized the amount of data that could be acquired over a wide range of speeds, which was critical for the validation of this concept.

**Figure 9:**
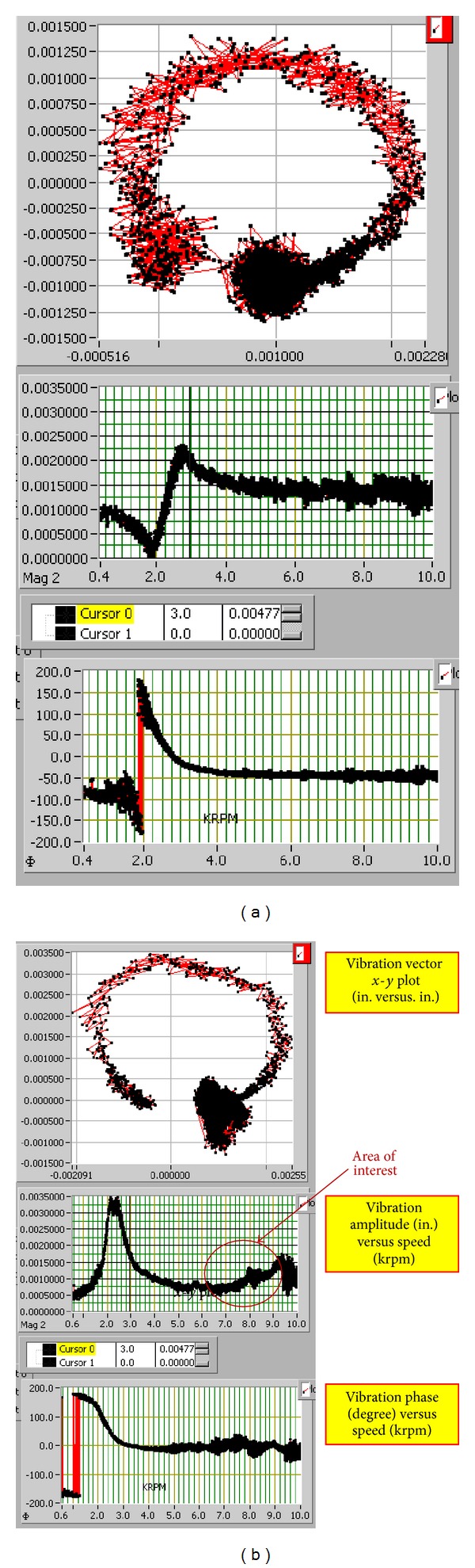
10 000 rpm multiple-cycle run comparison. (a) Baseline disk. (b) Notched disk.

**Figure 10:**
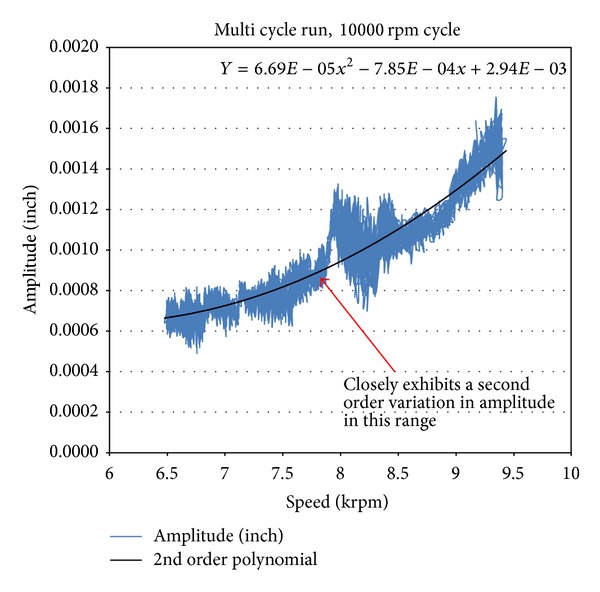
Detailed vibration amplitude plot for 10 000 rpm multiple-cycle run.

Figure 9 shows the results from this multiple-cycle test run for both the baseline and notched disks. The figure shows the analysis for the last cycle, starting at 10 000 rpm and ending at 0 rpm. As previously discussed, the presence of a crack is indicated if a second-order speed-dependent variation can be observed in the vibration amplitude as it is operated in the postcritical speed regime. In this test case, a speed-dependent rise was observed in the amplitude data for the notched disk. The area of interest showing the speed-dependent rise is highlighted within the circle of Figure 9(b). A curve-fit analysis, shown in Figure 10, was conducted on the vibration data in this region and it was found to closely follow a second-order polynomial fit, thus following the predicted behavior and potentially indicating the presence of the simulated crack.

Although the vibration data looked promising in this region, it was also observed that the second-order speed-dependent variation was not consistent over the entire range and that it appeared to reset itself at a speed of ~9500 rpm. Moreover, this relatively large amplitude variation was not consistently observed in other test runs, which cast some doubt on whether the effect was entirely due to the notch or to some other factor that is yet to be determined. However, this case yielded the best data so far and showed positive indications of being able to detect a defect such as a crack by analyzing the disk's vibration response as it is operated over a range of speeds. It is theorized that what made this case different, and more promising than other test runs, is that this test was conducted towards the end of the test series and many cycles had been placed on the disk prior to this test run. In addition, the disk experienced more loading because of the cyclical nature of the test profile. Overall, the results were promising and plans are in place to further investigate and refine this crack-detection technique as part of future studies.

## 4. Data Driven Anomaly Detection Technique

In addition to the experimental work previously described, parallel health monitoring assessments have been conducted using data mining algorithms in order to detect flaws, such as cracks, in a rotating engine disk. In this technique blade tip clearance data were acquired on an undamaged subscale engine disk using the same 10 000 rpm multi-cycle run profile that was used for the previously described vibration based technique. These baseline data were used to train three different data mining algorithms: Orca, Inductive Monitoring System (IMS), and One-Class Support Vector Machine (OCSVM) [12–15]. These algorithms were then used to analyze the blade tip clearance data that was acquired from a damaged disk which had a notch machined into it to simulate a crack in order to evaluate their performance and determine if the algorithms could detect the presence of the notch due to changes in the disks operating characteristics as measured by the blade tip clearance sensors.

As stated previously, three different techniques were investigated: Orca, Inductive Monitoring System (IMS), and One-Class Support Vector Machine (OCSVM). These methodologies provide a technique that can monitor the health of a system with fidelity by training the model to identify normal system parameters from abnormal ones. This is implemented by defining groups of consistent system parameter data using nominal system data. These training data are then used to model the system. Upon learning how the system should behave under nominal operating conditions, these models are then used to identify abnormal behavior, such as a crack or flaw in the disk which causes a change in the known operation of the system. 

### 4.1. Detection Algorithms

#### 4.1.1. Orca

Orca is an outlier detection algorithm which uses a Euclidean distance nearest neighbor based approach to determine outliers. For computational efficiency, it employs a modified pruning technique which allows it to perform in near linear time. For each point in the test data set, where a point is a row in the data set consisting of measurements taken at a single point in time, Orca calculates the nearest neighbor points from the reference data set. The output from Orca is a distance score which represents the average distance to its k-nearest neighbors; the more anomalous the point is the higher the score, since the nearest neighbors are farther away. More information about this algorithm can be found in [13].

#### 4.1.2. Inductive Monitoring System (IMS)

IMS is a cluster based modeling method. The algorithm is given a set of nominal data points and builds a model by agglomerative clustering of the data points. The resulting model is used to generate anomaly scores for new data. For each test data point, IMS finds its distance to the nearest cluster's boundary. The score that is reported is the sum of the squares of the distances from the test data point to the dimensional bounds of the closest cluster. If a data point falls entirely within the cluster bounds, the point is expected to represent normal behavior and it is assigned a score of zero. More information about IMS can be found in [12].

#### 4.1.3. One-Class Support Vector Machine (OCSVM)

OCSVM is a one-class nonlinear kernel based algorithm that maps the training data to a higher-dimensional feature space and then linearly separates nominal data from anomalies in that feature space. The idea is that such a model corresponds to a nonlinear model in the original data space but still maintains the benefit of a linear model in that it is guaranteed to return the model with the lowest error over the training set. The algorithm identifies a subset of the training data, called the “support vectors,” which is used to generate a hyperplane model. The anomaly score that is reported is the distance from the test data point to the hyperplane as measured in the feature space. More information on OCSVM can be found in [14, 15].

### 4.2. Analysis-Results

For the multiple-cycle test run previously shown in Figure 8 blade tip clearance data were recorded for both the undamaged and damaged, notched, disk. For the analysis, the 32 individual blade tip clearance measurements acquired for each revolution were utilized in training and evaluating the three anomaly detection algorithms. The undamaged disk's data was randomly divided in half. The first half was used for training of the algorithm and second half was used for validation. The means and standard deviations for all 32 channels were calculated for the training data and used to normalize both the training and testing data sets. 

**Figure 11:**
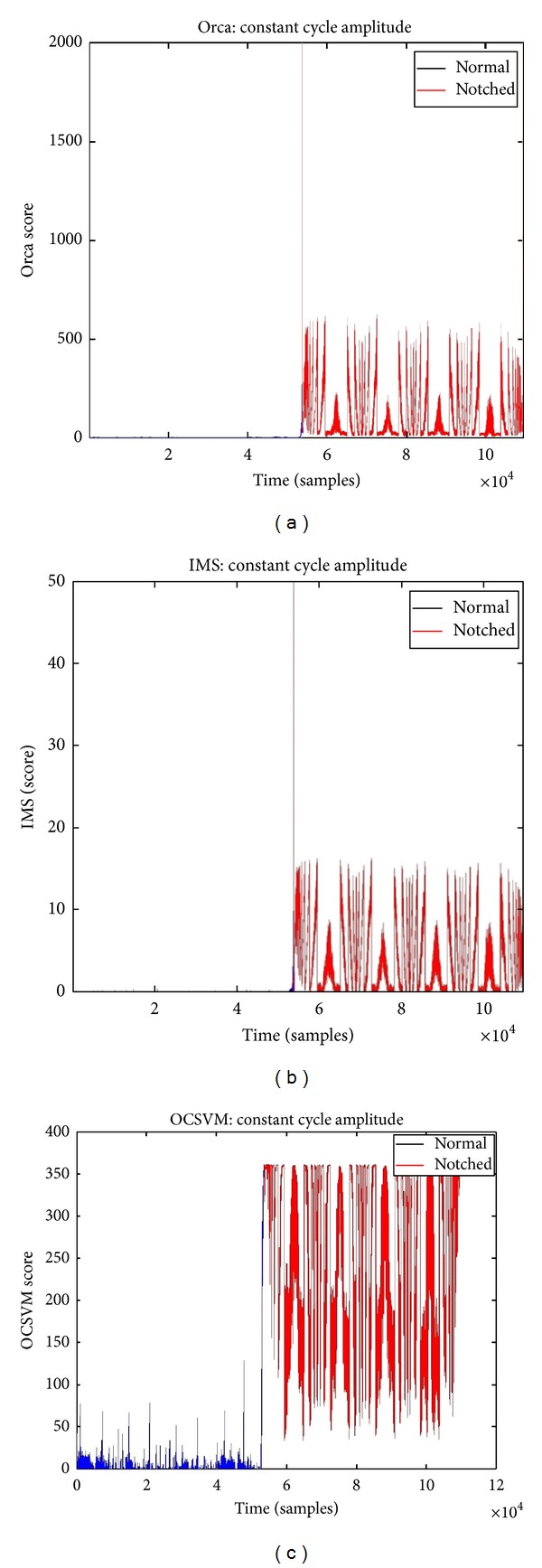
Results of the data driven anomaly detection techniques. (a) Orca. (b) IMS. (c) OCSVM.

The plots of Figure 11 are the global anomaly scores for each of the algorithms for both the validation undamaged data set and the damaged data set over time. The anomaly scores are portrayed as positive values and any nominal points are represented as zero. This was done to allow for similar comparison across the algorithms. It is important to note that OCSVM allows some nominal sample points in the training set to be classified as anomalous. This percentage is governed by the Nu parameter, which is set by the user, but for which we set currently at the default value of 10%. Due to this characteristic of the algorithm 10% of all nominal data tested may result in anomalous classification. In analyzing the effectiveness of the three techniques in Table 1 the threshold for correct detection was set to 90% to make the comparisons fair between all three algorithms, since OCSVM, in this case, was optimized for a correct detection of 90%.

Figure 11 shows all three algorithms and they appear to have performed quite well in differentiating between the nominal undamaged disk and the damaged, notched disk test runs. Orca and OCSVM seem to show slightly better performance across a few of the metrics. In Table 1 the metrics used for comparison are correct detection, false alarm rate, accuracy, and area under the Receiver Operating Characteristic (ROC) curve. The ROC curve is a plot of false positives versus true positives. The ideal curve is one that has a 90° bend in it shooting straight up with the false positive = 0 and holding the true positive = 1 across resulting in an area of 1.00. When fixing the false alarm rate at 5%, Orca and OCSVM both have 100% correct detection rate of the notched anomalous data, where IMS's correct detection rate is at 93%. When fixing a threshold so that the correct detection rate is 90%, all three show very good false alarm rates. An additional metric, that is independent of choosing a threshold, is measuring the area under the ROC curve. When comparing this metric across the three algorithms, all three methods are reporting very good areas with Orca and OCSVM doing slightly better than IMS. The results obtained showed that the detection algorithms are capable of predicting anomalies in the rotor disk with very good accuracy. Each detection scheme performed differently under the same experimental conditions and each delivered a different level of precision in terms of detecting a fault in the rotor.

## 5. Conclusion and Future Plans

The work presented in this paper represents a summary overview of the on-going research activities that are being conducted at the NASA Glenn Research Center for the development of new sensors and fault detection techniques for the structural health monitoring of rotating turbine engine components. The main focus of these efforts is to first develop and validate low technology readiness level (TRL) structural health monitoring concepts and then mature them for further use on more complicated aero engine hardware.

The microwave blade tip clearance sensor technology was found to be a promising option for use in structural health monitoring applications for gas turbine engines. The sensors have been successfully demonstrated on aero engine like hardware. The first generation 5.8 GHz sensors were used on a large axial vane fan and on a NASA Turbofan for the purposes of evaluating the sensor's ability to acquire blade tip clearance data on an aero engine size test article and blades. The second generation 24 GHz sensors were used to make basic blade tip deflection and low range clearance measurements on a simulated engine and on a small aero engine's compressor disk in order to evaluate the sensor's capability of making both clearance and timing measurements on small aero engine hardware. A ground test with these sensors installed on a full scale engine is being planned for the 2013-2014 timeframe. 

The vibration based crack detection experimental results showed that this technique has the potential and merit to be used for detecting cracks on a rotating disk. The data acquired from the 10 000 rpm multiple-cycle test run showed a speed-dependent rise in the vibration amplitude, indicating that a crack-induced shift in the disk's center of mass was present. Other test cases equally showed that the system was on the verge of being able to detect the simulated crack using this technique, but not all of the expected trends were consistently observed. Nevertheless, this technique looks very promising and appears to have the sensitivity to detect faults in the rotor disk system. However, additional work remains warranted to further validate and refine this crack detection concept.

Additional health monitoring was performed through combined experimental and data-driven anomaly detection techniques. Three different automatic data-driven detection algorithms, Orca, OCSVM, and IMS, were used. These techniques were utilized to analyze blade tip clearance data acquired from the rotor operating under undamaged and damaged conditions to check the viability of the detection methodology and to evaluate the performance of each approach. The results obtained showed that the detection algorithms are capable of predicting anomalies in the rotor disk with very good accuracy. Each detection scheme performed differently under the same experimental conditions and each delivered a different level of precision in terms of detecting a fault in the rotor. Overall rating showed that both the Orca and OCVSM performed better than the IMS technique, but in the end all of the algorithms showed promise and will be further refined for eventual use in detecting flaws in rotating components of an engine.

## Figures and Tables

**Table 1:** Multiple 10 000 rpm cycle test results.

	Algorithm	Correct detection rate	False alarm rate	Accuracy	Area under ROC
5% false alarm rate	Orca	100%	5%	97.55%	1.00
	IMS	93%	5%	94.02%	0.93
	OCSVM	100%	5%	97.55%	0.99

90% correct detection	Orca	90%	0.58%	94.62%	1.00
	IMS	90%	0.36%	94.73%	0.93
	OCSVM	90%	1.26%	94.28%	0.99
